# Origin of near to middle infrared luminescence and energy transfer process of Er^3+^/Yb^3+^co-doped fluorotellurite glasses under different excitations

**DOI:** 10.1038/srep08233

**Published:** 2015-02-04

**Authors:** Feifei Huang, Xueqiang Liu, Yaoyao Ma, Shuai Kang, Lili Hu, Danping Chen

**Affiliations:** 1 Key Laboratory of Materials for High Power Laser, Shanghai Institute of Optics and Fine Mechanics, Chinese Academy of Sciences, Shanghai 201800, PR China; 2 Graduate School of Chinese Academy of Sciences, Beijing 100039, PR China

## Abstract

We report the near to middle infrared luminescence and energy transfer process of Er^3+^/Yb^3+^ co-doped fluorotellurite glasses under 980, 1550 and 800 nm excitations, respectively. Using a 980 nm laser diode pump, enhanced 1.5 and 2.7 μm emissions from Er^3+^:I_13/2_→^4^I_15/2_ and I_11/2_→^4^I_13/2_ transitions are observed, in which Yb^3+^ ions can increase pumping efficiency and be used as energy transfer donors. Meanwhile, Yb^3+^ can also be used as an acceptor and intensive upconversion luminescence of around 1000 nm is achieved from Er^3+^:I_11/2_→^4^I_15/2_ and Yb^3+^: F_5/2_→^4^F_7/2_ transitions using 1550 nm excitation. In addition, the luminescence properties and variation trendency by 800 nm excitation is similar to that using 1550 nm excitation. The optimum Er^3+^ and Yb^3+^ ion ratio is 1:1.5 and excess Yb^3+^ ions decrease energy transfer efficiency under the two pumpings. These results indicate that Er^3+^/Yb^3+^ co-doped fluorotellurite glasses are potential middle- infrared laser materials and may be used to increase the efficiency of the silicon solar cells.

Over the past decades, Er^3+^/Yb^3+^ co-doped materials have attracted interest because of its usefulness for near to middle infrared emissions^1^,^2^,^3^,^4^. Erbium ion is an ideal luminescent center for 1.5 and 2.7 μm emissions, which correspond to the ^4^I_13/2_→^4^I_15/2_ and ^4^I_11/2_→^4^I_13/2_ transitions respectively^5^,^6^. The Er^3+^ doped fiber amplifier is one of the most important devices used in the 1.5 μm wavelength optical communication window^7^, and 2.7 μm emission also concerns researchers because of its possible applications in medicine, sensing, military countermeasures, and in light detection and ranging^8^,^9^,^10^. The absorption band of the Er^3+^:^4^I_15/2_→^4^I_11/2_ transition around 980 nm characterize weak ground state absorption and the sensitization of the Er^3+^ ions using Yb^3+^ ions increase the pumping efficiency^3^, as shown in Fig. 1(left). The Er^3+^/Yb^3+^ doped materials are used for effective energy transfer mechanisms for obtaining 1.5 and 2.7 μm emissions under 980 nm excitation, in which the Yb^3+^ ions are donors that transfer energy from the ^2^F_5/2_ level to the Er^3+^:^4^I_11/2_ level^11^,^12^. On the other hand, Yb^3+^ ions are acceptors when the Er^3+^/Yb^3+^ doped materials are excited using a 1550 nm Laser Diode (LD)^2^,^13^, as shown in Fig. 1(middle). The ions on the ^4^I_15/2_ ground state absorb two photons to the ^4^I_9/2_ levels. Then the ions on the ^4^I_9/2_ level decay radiatively or nonradiatively to the ^4^I_11/2_ level and the 1 and 2.7 μm emissions occur. The intensive 1000 nm upconversion luminescence converted from 1550 nm IR light in the Er^3+^/Yb^3+^ doped materials increase the efficiencies of Si solar cells because Si solar cells show highest efficiencies at 1000 nm wavelength, whereas only 60% of the visible light can be converted to electrons^14^,^15^. Meanwhile, the Er^3+^ ions can also be pumped directly to the ^4^I_9/2_ level under 800 nm excitation as shown in Fig. 1(right) and the luminescence properties should be similar to that under 1550 nm excitation theoretically. It is important to understand the luminous mechanism under different excitations for the Er^3+^/Yb^3+^ co-doped glasses in order to obtain more luminous information.

As host material for near to middle infrared emissions, glass attracts much research and development interest due to its ease of fabrication and its use as diode-pumped high-power solid state laser hosts, sensors, and optical amplifiers^16^,^17^,^18^,^19^. At present, attention have mainly been paid to the 2.7 μm emission of Er^3+^ doped fluoride glasses^20^ and 1 μm emission of Er^3+^/Yb^3+^ doped crystals^13^,^21^. As well known, so far most of works about 2.7 μm emission materials have been done in fluoride (ZBLAN) glasses. The glass family is the most stable one among all fluoride systems reported so far. In the past decade, Er doped and Er/Pr co-doped ZBLAN flbers have been developed for obtaining higher power output. But, the T_g_ of the ZBLAN is as low as 270°C which causes thermal effect. Additionally, because of the small value of ΔT, crystallization is an obstacle of fabricating high concentration ZBLAN fibers. These weaknesses limit the application of the ZBLAN in the future^22^. So it is important and challenging for researcher to find new mid-infrared materials. Fluorotellurite glass is a potential near to middle infrared laser material because it combines the advantages of fluoride and oxide glasses. Fluorotellurite glasses possess relatively low phonon energy among all the oxide glasses, a broad transmission window of 0.4~6 μm, and stable chemical and physical properties relative to fluoride glasses, such as easy fibering^23^. However, no works concern near to middle infrared emissions of Er^3+^/Yb^3+^ doped fluorotellurite glass excited under different wavelengths.

A new kind of fluorotellurite glass was prepared using AlF_3_- based glass modified with TeO_2_. Our previous work has reported the good thermal stability, low phonon energy and wide high transmittance of the glass^24^. In this study, near to middle infrared emissions of Er^3+^/Yb^3+^ doped glasses were measured under different excitations and the energy transfer processes between the two ions were determined. The optimum ratio of the two ions was chosen to obtain intensive 2.7 and 1 μm emissions. In addition, cross sections for the emissions and the energy transfer microparameters between the two ions were calculated.

## Experiments

The investigated glass has the following molar composition: 90(AlF_3_-YF_3_-CaF_2_-BaF_2_-SrF_2_-MgF_2_)–10TeO_2_–1ErF_3_-xYbF_3_ (x = 0, 0.5, 1, 1.5, 2, labelled as FEYx, respectively). All the samples were prepared using high-purity AlF_3_, YF_3_, CaF_2_, BaF_2_, SrF_2_, MgF_2_, TeO_2_, ErF_3_ and YbF_3_ powders. Well-mixed 25 g batches of the samples were placed in platinum crucibles and were melted at about 950°C for 30 min. Then the melts were poured onto a preheated copper mold and annealed in a furnace at around the glass transition temperature. The annealed samples were fabricated and polished to 20 mm × 15 mm × 1 mm dimensions for the optical property measurements.

The characteristic temperatures (temperature of glass transition T_g_ and temperature of the onset of the crystallization peak T_x_) of the samples were determined using a NetzschSTA449/C differential scanning calorimeter at the heating rate of 10 K/min. The densities and refractive indices of the samples were measured using the Archimedes method, with distilled water as the immersion liquid and the prism minimum deviation method respectively. Furthermore, the absorption spectra were recorded using a Perkin-Elmer Lambda 900 UV/VIS/NIR spectrophotometer at the range of 300 nm to 2000 nm, and the emission spectra were measured using a Triax 320 type spectrometer (Jobin-Yvon Co., France). All the measurements were performed at room temperature.

## Results

**Figure 2:**
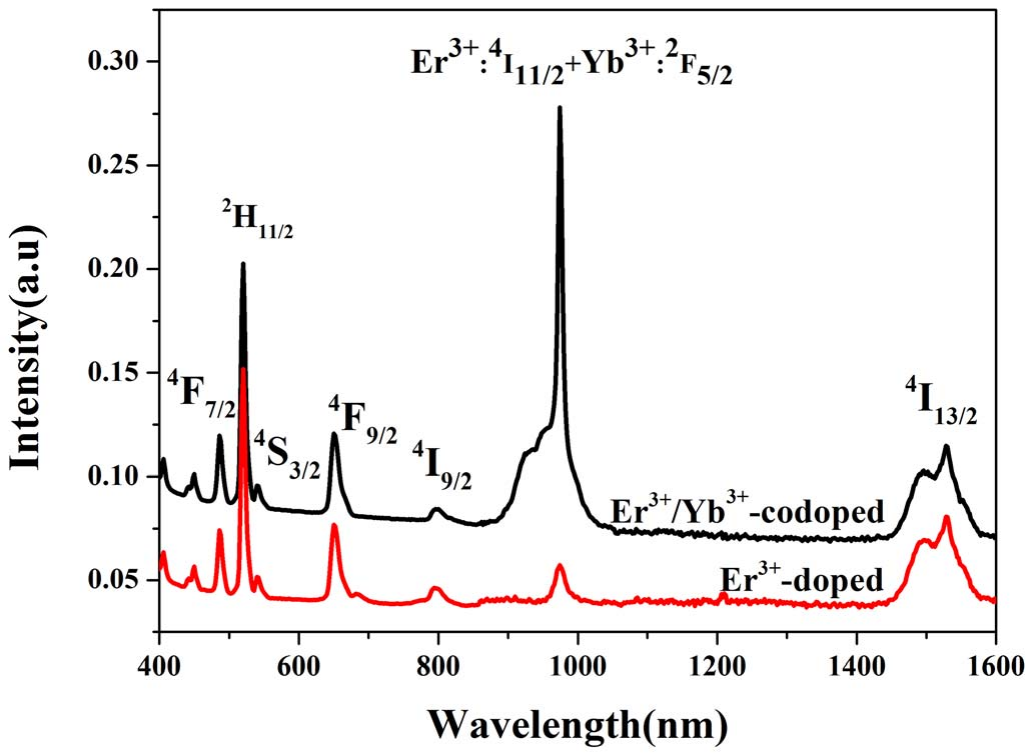
Absorption spectra of the FE and FEY1.5 samples.

Fig. 2 shows the absorption spectra of the Er^3+^- doped and Er^3+^/1.5Yb^3+^ co-doped samples at room temperature in the wavelength region of 400 nm to1600 nm. The introduction of 1.5 mol% Yb^3+^ greatly enhances the absorption coefficient at around 980 nm, resulting in the efficient absorption of the pump source at around 980 nm. The radiative transition parameters within the 4f^n^ configuration of the Er^3+^ ions can be analyzed using the Judd-Ofelt (J-O) theory and can be accurately measured using absorption spectra^25^,^26^. The J-O parameters and radiative transition parameters (spontaneous transition probability *A*, branching ratio β, and calculated lifetime τ) for the Er^3+^:^4^I_11/2_→^4^I_13/2_ transition of the present FE and FEY1.5 samples have been calculated, and are shown in Table 1. Ω_2_ parameters indicate the amount of the covalent bond, and are strongly dependent on the local environment of the ion sites, whereas the Ω_6_ parameter is related to the overlap integrals of the *4f* and *5d* orbits^27^,^28^. The higher Ω_2_ of the codoped sample indicates a higher covalency and lower symmetry. The spectroscopic quality factor i.e. Ω_4_/Ω_6_ is an important parameter to predict the stimulated emission in a laser active host^29^. The spectroscopy quality factor (1.31) in the co-doped sample is much larger than those reported in fluoride glasses^30^, indicating that the co-doped sample is a favorable optical material. The predicted spontaneous emission probability for the 2.7 μm emission is larger in the co-doped sample, which provides a better opportunity to obtain laser actions^31^.

**Figure 3:**
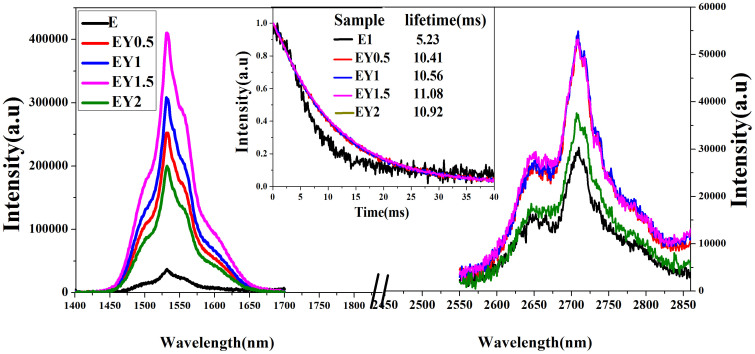
1.5 μm (left) and 2.7 μm (right) emissions of the present samples under 980 nm excitation. The inset is the decay lifetime of the 1.5 μm emission.

Figure 3 shows the emission spectra around 1.5 and 2.7 μm and the measured decay lifetimes of the 1.5 μm emission before and after Er^3+^ co-doped Yb^3+^ under 980 nm excitation. The intensities of the two emissions initially increase as Yb^3+^ ions increase, whereas the excess Yb^3+^ ions (>1.5 mol %) reduce the intensities. The 1.5 and 2.7 μm emissions originate from the Er^3+^:^4^I_13/2_→^4^I_15/2_ and ^4^I_11/2_→^4^I_13/2_ transitions, and the intensities of the two emissions are enhanced 12 and 2 times, respectively, when the ratio of the Er^3+^ and Yb^3+^ ions is 1:1.5. The Yb^3+^ ions positively affect the 1.5 and 2.7 μm emissions of Er^3+^ ions under 980 nm excitation, and the energy transfer process is from the Yb^3+^:^2^F_5/2_ to the Er^3+^:^4^I_11/2_ level. The change of the decay lifetimes with the Yb^3+^ contents coincides with those of the emissions. The lifetime is an important factor for potential laser materials. The full width at half maximum (FWHM)^32^ determines 1.5 μm laser materials. The larger bandwidth of this transition is suitable for tunable lasers that deliver relatively constant power over a wide wavelength range. The FWHM value in the EY1.5 glass in this study is about 55 nm, which is not only higher than those of silicate (34.8 nm)^33^ and phosphate (46.0 nm)^33^ but also higher than those of pure fluoride ZELAG (46 nm)^34^ and tellurite glasses (53 nm)^35^. The emission cross section is an important parameter for 2.7 μm emission which can be calculated as^10^,^36^,^37^
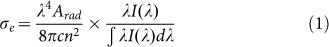
where λ is the wavelength. A_rad_ is the spontaneous transition probability. I(λ) is the emission spectrum, n and c are the refractive index and light speed in vacuum respectively. The emission cross section of the ^4^I_11/2_→^4^I_13/2_ transition of the EY1.5 sample is calculated to be 8.3 × 10^−21^ cm^2^, which is higher than those of Er^3+^ doped ZBLAN glass (5.4 × 10^−21^ cm^2^)^29^, chalcohalide glass (6.6 × 10^−21^ cm^2^)^29^, fluorophosphate glass (7 × 10^−21^ cm^2^)^38^, and tellurite glass (6.1 × 10^−21^ cm^2^)^39^.

**Figure 4:**
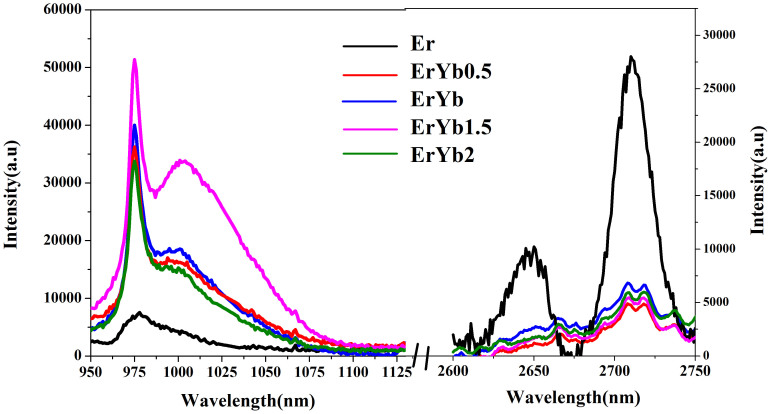
1 μm (left) and 2.7 μm (right) emissions of the samples under 1550 nm excitation.

Figure 4 shows the emission spectra around 1 and 2.7 μm before and after Er^3+^ co-doped Yb^3+^ under 1550 nm excitation. The upconversion luminescence bands centered at 980 nm is a two- photon process and originate from the Er^3+^:^4^I_11/2_→^4^I_15/2_ and Yb^3+^:^4^F_5/2_→^4^F_7/2_ transitions. After introducing Yb^3+^, the line shapes of the emission from the co-doped samples significantly change and are similar to those recorded from other materials containing Tb^3+^/Yb^3+^ and Pr^3+^/Yb^3+^ 40,41, which indicates that the emission is probably due to the transition in the Yb^3+^ ions and the energy transfer process from the Er^3+^:^4^I_11/2_ to the Yb^3+^:^2^F_5/2_ level. The intensity of the emission is highest when the Er^3+^ and Yb^3+^ ratio is 1:1.15. The energy transfer process cannot be efficiently performed with excess Yb^3+^ ion content the content. The obvious 2.7 μm emission is observed in the Er^3+^ singly doped sample and it is hardly to be obtained in the co-doped samples which can be explained by the energy transfer from the Er^3+^:^4^I_11/2_ level to the Yb^3+^:^2^F_5/2_ level.

**Figure 5:**
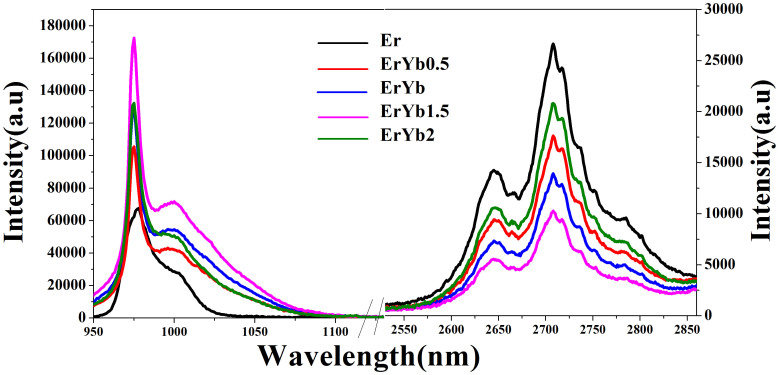
1 μm (left) and 2.7 μm (right) emissions of the samples under 800 nm excitation.

Figure 5 shows the emission spectra around 1 and 2.7 μm before and after Er^3+^ co-doped Yb^3+^ under 800 nm excitation. The similar phenomenon has been observed as that under 1550 nm excitation. The 1 μm is enhanced significantly in the co-doped samples and the 2.7 μm emission is decreased with the increasing Yb^3+^ ions when the Yb^3+^ content is below 1.5 mol %, which demonstrate the Yb^3+^ ions accept the energy from the Er^3+^ ions and the 1 μm emission mainly comes from the Yb^3+^:^4^F_5/2_→^4^F_7/2_ transition. Figure 5 also shows the energy transfer process can proceed efficiently when the ratio of the Er^3+^ and Yb^3+^ ions is 1:1.5.

## Discussions

As discussed above, the Yb^3+^:^2^F_5/2_ level can transfer energy to the Er^3+^:^4^I_11/2_ and the backward process can also occur. If energy of the emission transition of one RE^3+^ ion (called the donor) is equal or close to the energy of the absorption transition of the other RE^3+^ ion (called the acceptor). Energy transfer between rare earth ions can occur according to the Forster and Dexter theory^42^,^43^,^44^. The absorption cross section of the Er^3+^:^4^I_11/2_→^4^I_15/2_ and Yb^3+^:^4^F_7/2_→^4^F_5/2_ transitions can be deduced by the Beer-Lambert equation^43^
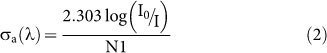
where 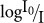 is the absorptivity from absorption spectrum, l is the thickness of the glass and N is the ion density.

The emission cross section can be obtained by using the McCumber equation^3^: 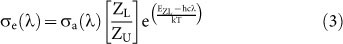
where h is Planck's constant, K_B_ is the Boltzmann constant, T is the temperature, E_zl_ is the ground state manifold and the lowest stark level of the upper manifolds and Z_u_ and Z_l_ are partition functions of the lower and upper manifolds.

**Figure 6:**
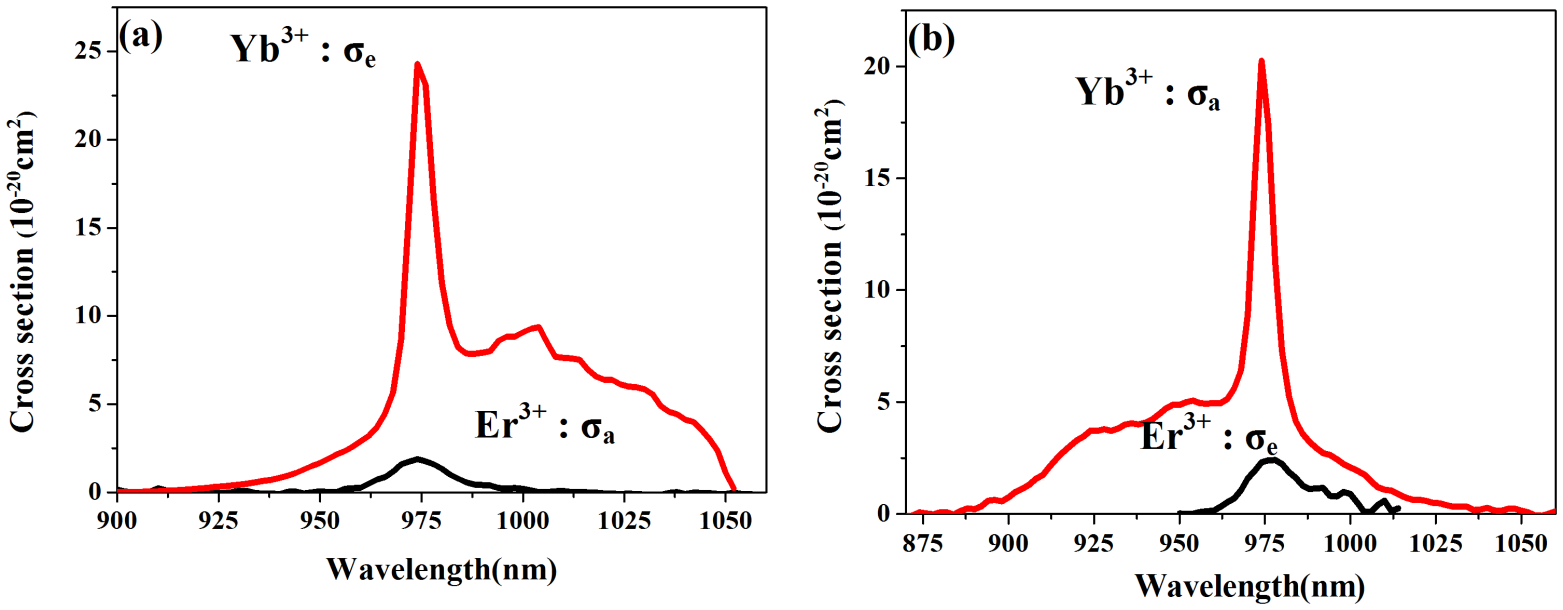
Absorption and emission cross sections of the Yb^3+^ and Er^3+^ ions.

Fig. 6 shows the absorption and emission cross sections of the Yb^3+^ and Er^3+^ ions. Figure (a) describes the energy transfer process from Yb^3+^ to Er^3+^, which corresponds to the results under 980 nm excitation, and (b) describes the reverse process which corresponds to the results under 1550 and 800 nm excitations. The overlap between σ_a_ and σ_e_ is quite large, therefore, efficient energy transfer can be expected between the two ions.

Based on the obtained absorption cross section of the donor and the emission cross section of the acceptor, the probability rate of the energy transfer between the donor and the acceptor can be described as 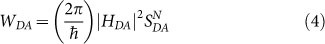
where 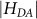 is the matrix element of the Hamiltonian perturbation between the initial and final states in the energy transfer process, and 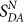 is the overlap integral between the *m*-phonon emission line shape of the donor ions (D) and the *k*-phonon emission line shape of the donor ions (A). In the case of weak electron-phonon coupling, 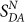 can be approximated as 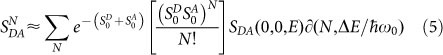
where S_DA_ (0, 0, E) represents the overlap integral between the zero-phonon line shape of the donor emission ions and the absorption of the acceptor ions, and S_0_^D^, and S_0_^A^ are the Huang-Rhys factors of the donor and acceptor ions, respectively. The probability rate of the energy transfer can be obtained using the following direct transfer equation:
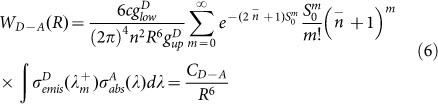
where C_D-A_ is the energy transfer coefficient, and R is the distance of separation between the donor and acceptor, and the critical radius of the interaction can be obtained by the equation 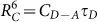, and τ_D_ is the intracenter lifetime of the excited level of the donor. The expression for direct transfer (D-A) is expressed as:
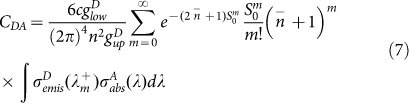
The microparameters of energy transfer from Yb^3+^:^2^F_5/2_ to the Er^3+^:^4^I_11/2_ and the reverse process are calculated using Eqs (4)–(7). The values are 2.06 × 10^−39^ and 2.12 × 10^−39^ cm^6^/s, respectively, and they both are independent of phonon in the quasiresonant process. These results show that the high energy transfer process efficiency between the two ions and the direction of the process are dependent on the excitations. The Er^3+^/Yb^3+^ co-doped fluorotellurite glasses can be used to obtain 1.5 and 2.7 μm emissions under 980 nm excitation and 1000 nm upconversion luminescence under 1550 nm excitation.

## Conclusion

In conclusion, Er^3+^/Yb^3+^ co-doped fluorotellurite glasses are prepared, and their near to middle infrared emissions under different excitations were investigated. The 1.5 and 2.7 μm emissions are effectively enhanced by the presence of Yb^3+^ ions under 980 nm excitation. The FWHM of the 1.5 μm emission is as high as 55 nm and the emission cross section of the 2.7 μm emission reaches 8.3 × 10^−21^ cm^2^. Under 1550 nm excitation, the Yb^3+^ ions accept the energy from the Er^3+^ ions, and the upconversion luminescence centered at λ = 980 nm from the co-doped samples is enhanced. The optimum Er^3+^ and Yb^3+^ ion ratio in this system is 1:1.5, under different excitations. The energy transfer microparameters from the Er^3+^ to Yb^3+^ and the reverse process are calculated to be 2.06 × 10^−39^ and 2.12 × 10^−39^ cm^6^/s, respectively. Therefore, the 1.5 μm emission is useful optical communication window, 2.7 μm has possible applications in medicine, sensing, and military countermeasures, and 980 nm upconversion luminescence corresponds to the most efficient absorption wavelength of Si solar cells and can be used to increase their efficiency.

## Figures and Tables

**Figure 1:**
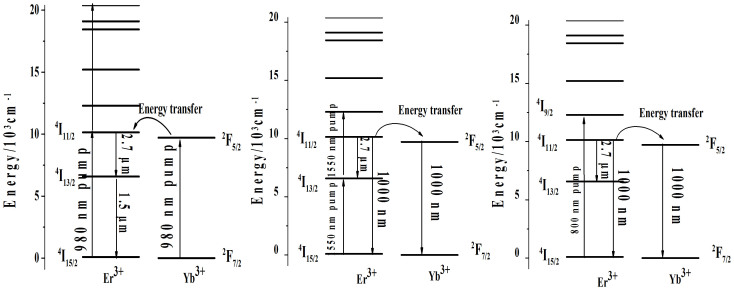
Energy levels of the Er^3+^ and Yb^3+^ ions and energy transfer processes under 980 (left), 1550 (middle), and 800 (right) nm excitations.

**Table 1:** The J-O parameters and radiative transition parameters (spontaneous transition probability *A*, branching ratio β, and calculated lifetime τ) for the Er^3+^:^4^I_11/2_→^4^I_13/2_ transition of the FE and FEY1.5 samples

Glass	Ω_2_ (×10^−20^ cm^2^)	Ω_4_ (×10^−20^ cm^2^)	Ω_6_ (×10^−20^ cm^2^)	Ω_4_/Ω_6_	δ (×10^−6^)	*A* (S^−1^)	β (%)	τ (ms)
FE	2.74	1.12	0.96	1.16	0.25	20.95	19.08	9.52
FEY1.5	2.85	1.37	1.05	1.31	0.31	21.15	18.64	8.82
